# Anti‐Phosphatidylserine/Prothrombin Antibodies Identify a Distinct Form of ITP

**DOI:** 10.1002/jha2.70154

**Published:** 2025-10-04

**Authors:** Sofia Camerlo, Massimo Radin, Giorgio Rosati, Melissa Padrini, Barbara Montaruli, Isabella Russo, Cristina Barale, Alice Barinotti, Irene Cecchi, David Galarza, Fulvio Pomero, Marco De Gobbi, Savino Sciascia, Alessandro Morotti

**Affiliations:** ^1^ Department of Clinical and Biological Sciences University of Turin Turin Italy; ^2^ Department of Internal Medicine M. and P. Ferrero Hospital Verduno Italy; ^3^ Department of Clinical and Biological Sciences, and ASL Città di Torino University Center of Excellence on Nephrologic, Rheumatologic and Rare Diseases (ERKNet, ERN‐Reconnet and RITA‐ERN Member) with Nephrology and Dialysis Unit and Center of Immuno‐Rheumatology and Rare Diseases (CMID), Coordinating Center of the Interregional Network for Rare Diseases of Piedmont and Aosta Valley, San Giovanni Bosco Hub Hospital Turin Italy; ^4^ Pathology Division AUO Mauriziano Turin Italy; ^5^ Department of Rheumatology Hospital Del Mar Barcelona Barcelona Spain

**Keywords:** antiphospholipid syndrome, immune thrombocytopenia, phosphatidylserine/prothrombin antibodies

## Abstract

**Introduction:**

Immune thrombocytopenia (ITP) and antiphospholipid syndrome (APS) are distinct autoimmune disorders with clinical intersections. While APS is marked by thrombosis and pregnancy complications, ITP typically presents as isolated low platelet counts with bleeding risk. Thrombocytopenia can also occur in APS, and a subset of ITP patients test positive for antiphospholipid antibodies (aPL).

**Discussion:**

This study examined aPL in 90 ITP patients and compared them to 132 APS patients. Among the ITP group, 18.3% were aPL‐positive, with lupus anticoagulant most common; 12% had anti‐phosphatidylserine/prothrombin (aPS/PT) antibodies. In the APS cohort, 16% exhibited thrombocytopenia (< 100,000/µL), with a mean platelet count of 44,000/µL, and 71% of these were positive for aPS/PT. Platelet levels varied significantly among ITP aPL‐negative, ITP aPL‐positive and APS‐ITP groups (*p* < 0.001). aPL positivity was linked to less severe thrombocytopenia but affected treatment approaches, showing differences in first‐line (*p* = 0.034) and second‐line (*p* = 0.025) therapies. Patients with ITP secondary to APS had a higher mean platelet count compared to aPL‐positive ITP patients and aPL‐negative ITP patients.

**Conclusion:**

Screening for aPL and aPS/PT is vital to identify an ITP subset with milder thrombocytopenia and increased thrombotic risk, and may guide therapeutic decisions such as between thrombopoietin receptor agonists and SYK inhibitor.

**Trial Registration:**

The authors have confirmed clinical trial registration is not needed for this submission.

## 1 Introduction

Antiphospholipid syndrome (APS) [1] and immune thrombocytopenia (ITP) [2] are two distinct autoimmune disorders, but they may be closely related and can co‐occur in some patients. ITP can develop as a secondary condition to APS, and conversely, thrombocytopenia is included in the classification criteria for APS [3]. This overlap suggests shared immune mechanisms between ITP and APS, underscoring the need for thorough evaluation in thrombocytopenia cases. APS diagnosis relies on clinical features and the detection of specific prothrombotic antibodies, including lupus anticoagulant (LA), IgG/IgM anti‐cardiolipin (aCL) and anti‐beta‐2 glycoprotein I (aB2GP1) antibodies [3]. In addition to these conventional diagnostic markers, recent research has recognised a set of supplementary antibodies known as ‘extra‐criteria’ antiphospholipid antibodies (aPL) [4]. These include IgG and IgM anti‐phosphatidylserine/prothrombin (aPS/PT) antibodies, which have been shown to be relevant in APS diagnosis, particularly in patients who test negative for traditional markers but show clinical symptoms suggestive of the syndrome [4, 5, 6]. Emerging evidence indicates that aPS/PT positivity may identify APS patients at higher risk for thrombosis or pregnancy complications, underscoring the need for better risk stratification [7, 8, 9, 10]. The association between aPS/PT positivity and thrombocytopenia has already been documented in patients with APS [11], and a single case report has postulated a link between aPS/PT positivity and microangiopathy [12]. However, this correlation remains unstudied in ITP and, to our knowledge, unreported. aPS/PT positivity may signal a more severe phenotype, potentially increasing thrombotic risk, especially with thrombopoietin (TPO) receptor agonist treatment [13, 14]. As ITP treatments increasingly intersect with thrombotic risk, refined patient stratification is crucial for guiding therapy. APS involves clot formation in veins and arteries, often causing complications like DVT, stroke or pregnancy loss due to aPL‐induced abnormal clotting [15]. It is therefore recommended that patients with ITP receive an accurate diagnosis to assess and manage the potential risk of thrombosis, which may be increased depending on therapeutic choices, such as the use of TPO mimetics [16]. Thrombocytopenia in APS, resembling ITP, may offer insights into their relationship. Understanding the role of conventional and extra‐criteria aPL in ITP is key to improving diagnosis and treatment. This study examines aPL prevalence in primary ITP and aims to identify a distinct ITP subset defined by conventional aPL and aPS/PT antibodies, with overlapping features of APS‐related ITP. It also evaluates the role of advanced antibody testing in guiding care.

## 2 Methods

### 2.1 Patients

A total of 222 patients (90 with ITP and 132 with APS) attending the San Luigi Gonzaga Hospital and the San Giovanni Bosco Hospital (Turin, Italy) constituted the initial cohort from which the study population was derived. Clinical data were collected retrospectively from medical charts.

Inclusion criteria were:

a.1) Either diagnosis of APS or persistent aPL positivity [3]
a.2) Diagnosis of ITP per international guidelines [17, 18]
b) ≥ 1‐year follow‐up
c) In the APS‐cohort only patients with < 100,000/µL were included in the statistical analysis and compared to ITP cohort.

Thrombocytopenia severity is referred as: mild (< 100,000/≥ 50,000 µL), moderate (< 50,000/≥ 20,000 µL) and severe (<20,000 µL). The study was approved by the ethical committee (code #80/2023); all patients provided informed consent.

### 2.2 Data Collection

Demographic, laboratory and clinical data were collected every 6 months or during new clinical events. Arterial risk factors included diabetes, hypertension, hypercholesterolemia, obesity, smoking and family history. Venous risk factors included hormone therapy, pregnancy, malignancy, family history and thrombophilia (e.g., antithrombin, protein C/S deficiencies, factor V Leiden, prothrombin G20210A, hyperhomocysteinemia, high factor VIII). Malignancy was defined as active at assessment.

### 2.3 Autoantibody Detection

The aPL profile included LA, aCL, anti‐ß2GPI and aPS/PT antibodies. Plasma samples were tested according to the recommended criteria from the International Society on Thrombosis and Haemostasis (ISTH) Subcommittee on Lupus Anticoagulant/Phospholipid‐Dependent Antibodies [19]. The aCL, anti‐ß2GPI and aPS/PT antibodies were detected by ELISA as described previously [20]. Cut‐off values provided were determined by manufacturer recommendations and clinically relevant values were considered as: aCL and anti‐ß2GPI > 40 U/mL; aPS/PT IgG > 30 U/mL, IgM > 40 U/mL. All antibodies were revaluated at 12 weeks. When indicated in the manuscript, aPL positivity was defined as the presence of at least one positive result for conventional antiphospholipid antibodies and/or anti‐phosphatidylserine/prothrombin antibodies. This definition includes patients who were positive for aPS/PT only, even in the absence of conventional aPL. It is important to note that in ITP patients, aPLs were tested prior to the administration of intravenous immunoglobulins to prevent potential interference with the results. Patients who received IVIg treatment prior to aPL testing, as well as those with a second negative test at 12 weeks, were excluded from the analysis.

### 2.4 Statistical Analysis

Pearson's Chi‐square test (*χ* ^2^) and Fisher's exact test were used for the comparison of categorical variables. For continuous variables, unpaired *t*‐tests and one‐way analysis of variance (ANOVA) were applied, as appropriate. Multivariable linear regression was used to assess the independent association between variables of interest, adjusting for potential confounders. All statistical analyses were performed using Jamovi software, version 2.3.28.

## 3 Results

### 3.1 ITP Cohort Assessment

**FIGURE 1:**
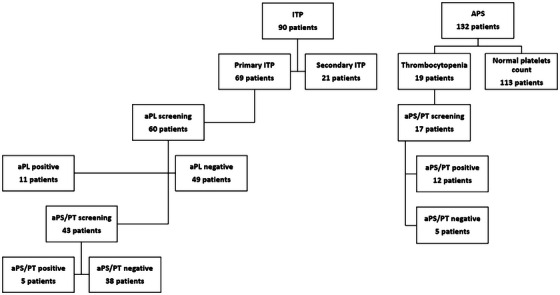
Overview of the screening and selection process for patients included in the study. The flow chart of patient's enrolment and analysis is indicated for each individual group.

A total of 90 patients were included: 76.7% had primary and 23.3% secondary ITP. Sixty individuals with no other identifiable causes of ITP were screened for aPL (LA, aCL IgG and IgM, aβ2GPI IgG and IgM) (Figure 1), 11 (18.3%) were positive for aPL. A total of 72.2% (8/11) were positive for LA. A minority of patients (18.2%) exhibited triple positivity, while the majority demonstrated single (45.4%) or double positivity (36.4%). A comprehensive aPL profile, demographic and laboratory characteristics are presented in Table 1. We compared the mean platelet count between LA‐positive and LA‐negative patients. LA‐positive patients showed a higher mean platelet count (32,250/µL ± 15,673) compared to LA‐negative patients (15,000/µL ± 7,000); however, this difference did not reach statistical significance (*p* = 0.107). We assessed whether the aPL profile (single, double or triple) correlated with thrombocytopenia severity; no statistically significant association was found (*p* = 0.374). When comparing aPL‐negative patients (46 patients) and aPL‐positive patients (14 patients, including those positive for at least one aPL, including isolated aPS/PT positivity), the mean platelet counts were 21,130/µL vs. 27,429/µL (*p* = 0.319). Notably, a statistically significant difference was observed in the distribution of thrombocytopenia severity between the two groups (severe thrombocytopenia: 65.2% vs. 28.6%; moderate: 19.6% vs. 64.3%; mild: 15.2% vs. 7.1%; *p* = 0.006).

**TABLE 1:** Comparison of demographic, laboratory, and treatment characteristics among aPL‐negative ITP patients, aPL‐positive ITP patients, and ITP secondary to APS.

	ITP aPL negative *n* = 46	ITP aPL positive *n* = 14	ITP secondary to APS *n* = 19
**Demographic characteristics**			
Mean age at data collection (years) +/− ds	57.1 years ± 18.8	46.9 years ± 19.9	42.0 years +/− 14.2
Female, *n* (%)	31 (67.4%)	6 (42.9%)	13 (68.4%)
Male, *n* (%)	15 (32.6%)	8 (57.1%)	6 (31.6%)
**Laboratory characteristics**			
PLTS count (/µL)	21.130 ± 21.575	27.429 ± 16.327	43.895 +/− 21.947
Hypo C3	6.3%	33.3%	23.5%
Hypo C4	25.0%	33.3%	17.6%
**aPL profile**			
Conventional aPL		11 (78.6%)	19 (100%)
LA, *n* (%)		8 (72.2%)	16 (84.2%)
IgG aCL, *n* (%)		4 (36.4%)	9 (47.4%)
IgM aCL, *n* (%)		2 (18.2%)	4 (21.1%)
IgG aB2GPI, *n* (%)		2 (18.2%)	10 (52.6%)
IgM aB2GPI, *n* (%)		4 (36.4%)	6 (31.6%)
		*n* = 11	*n* = 17
aPS/PT, *n* (%)		5 (45.5%)	12 (70.6%)
IgG aPS/PT, *n* (%)		2 (18.2%)	8 (42.1%)
IgM aPS/PT, *n* (%)		4 (36.4%)	9 (47.4%)
**Treatment**			
No treatment, *n* (%)	6 (13.0%)	4 (28.6%)	8 (42.1%)
Only corticosteroids, *n* (%)	10 (21.7%)	7 (50.0%)	9 (47.4%)
Corticosteroids and IVIG, *n* (%)	30 (65.2%)	3 (21.4%)	2 (10.5%)
Second or further lines of therapy, *n* (%)	21 (45.7%)	6 (42.9%)	2 (10.5%)
Rituximab, *n* (%)	5 (10.9%)	2 (14.3%)	2 (10.5%)
TPO‐mimetics, *n* (%)	20 (43.5%)	6 (42.9%)	1 (5.3%)
Splenectomy, *n* (%)	7 (15.2%)	1 (7.1%)	0
Fostamatinib, *n* (%)	3 (6.5%)	0	0
Other immunosuppressive drugs, *n* (%)	3 (6.5%)	2 (14.3%)	1 (5.3%)
**APS‐related clinical features**			
Thrombosis history, *n* (%)			17 (89.5%)
Pregnancy morbidity, *n* (%)			3 (15.8%)

To isolate the effect of conventional aPL (excluding aPS/PT), we compared aPL+/aPS/PT− patients with those negative for all aPL. No significant difference in mean platelet count was observed (26,222 vs. 21,130/µL; *p* = 0.446). Although not statistically significant (*p *= 0.075), severe thrombocytopenia was more frequent in the double‐negative group (65.2% vs. 33.3%), while moderate thrombocytopenia was more common in the aPL+/aPS/PT– group (55.5% vs. 19.6%). These findings suggest limited impact of conventional aPL on disease severity. The association between aPS/PT positivity and thrombocytopenia severity was confirmed in the subgroup of 43 patients tested for aPS/PT, with a positivity rate of 11.6% (5/43). Among these, two were positive for both conventional aPL and aPS/PT, while three had isolated aPS/PT positivity. Demographic and laboratory characteristics are shown in Table 2. aPS/PT‐positive patients had a higher mean platelet count than aPS/PT‐negative patients (29,600/µL vs. 21,868/µL). Notably, 80% of aPS/PT‐positive patients had moderate thrombocytopenia, versus 15.8% of aPS/PT‐negative patients. In contrast, 65.8% of aPS/PT‐negative patients had severe thrombocytopenia, compared to 20% in the aPS/PT‐positive group. This difference in thrombocytopenia severity was statistically significant (*p* = 0.006), supporting the hypothesis that aPS/PT positivity may define a distinct ITP phenotype, characterised by a milder presentation. Correlation analyses between aPS/PT IgG/IgM titres and platelet counts or thrombocytopenia severity showed no significant associations (*p* = 0.417 and *p* = 0.742, respectively). Regarding treatment, 79 of 90 patients (87.8%) received first‐line therapy: 27 (30.0%) with corticosteroids alone and 52 (57.8%) with corticosteroids plus IVIG. During follow‐up, 50 patients (55.6%) required second‐line therapy, 21 (23.3%) third‐line and 9 (10.0%) fourth‐line or additional treatments. Second‐line or later therapies included TPO receptor agonists (44 patients, 48.9%), rituximab (18, 20.0%), splenectomy (14, 15.6%) and fostamatinib (3, 3.3%). Stratified analyses by ITP subtype (primary vs. secondary) and aPL status (positive vs. negative) showed no significant differences in first‐line therapy (86.9% vs. 89.4%, *p* = 0.772), IVIG use (55.1% vs. 73.7%, *p* = 0.147) or second‐line treatments (55.1% vs. 63.2%, *p* = 0.534). Similarly, no differences were observed between aPL‐negative and aPL‐positive patients in overall treatment need (87.0% vs. 71.4%, *p* = 0.178). However, aPL‐negative patients were significantly more likely to receive corticosteroids plus IVIG (65.2% vs. 21.4%, *p* = 0.003). No differences were seen in frequency or type of second‐line treatments (46.7% vs. 46.1%, *p* = 0.857).

**TABLE 2:** Comparison of demographic, laboratory, and treatment characteristics between aPS/PT‐positive and aPS/PT‐negative ITP patients.

	ITP aPS/PT negative *n* = 38	ITP aPS/PT positive *n* = 5
**Demographic characteristics**		
Mean age at data collection (years) +/− ds	54,9,1 years ± 19,8	58,8 years ± 20,4
Female, *n* (%)	24 (63.2%)	2 (40.0%)
Male, *n* (%)	14 (36.8%)	3 (60.0%)
**Laboratory characteristics**		
PLTS count (/µL)	21.868 ± 22.715	29.600 ± 16.832
aPL profile		
IgG aPS/PT, *n* (%)		2 (40.0%)
IgM aPS/PT, *n* (%)		4 (80.0%)
Conventional aPL, *n* (%)	6 (15.8%)	2 (40%)
**Treatment**		
No treatment, *n* (%)	7 (18.4%)	1 (20.0%)
Only corticosteroids, *n* (%)	10 (26.3%)	3 (60.0%)
Corticosteroids and IVIG, *n* (%)	21 (55%)	1 (20.0%)
Second or further lines of therapy, *n* (%)	20 (52.6%)	2 (40.0%)

### 3.2 APS Cohort Assessment

In the cohort of 132 patients with APS, 21 subjects (15.9%) had a concomitant diagnosis of thrombocytopenia, defined as a platelet count below 130,000/µL, in accordance with the 2023 ACR/EULAR classification criteria [3]. However, in accordance with the inclusion criteria for this study and the definition of ITP, only 19 patients with a platelet count below 100,000/µL were included (Figure 1). A total of 78.5% (15/19) of patients were diagnosed with primary APS, while 21.5% (4/19) had secondary APS associated with systemic lupus erythematosus (SLE). The mean age of patients with ITP secondary to APS was 42.0 ± 14.2 years, with 68.4% of the cohort being female (Table 1). The mean platelet count was 43,895/µL, with a standard deviation of ± 21,947/µL. No statistically significant differences were observed in platelet counts between primary and secondary APS patients (46,867/µL vs. 32,750/µL; *p* = 0.265). Similarly, the severity of thrombocytopenia did not differ significantly between groups (p = 0.825). The aPL profile in APS patients with thrombocytopenia revealed a high prevalence of LA (84.2%), followed by aβ2GPI IgG (52.6%), aCL IgG (47.4%), aβ2GPI IgM (31.6%) and aCL IgM (21.1%). Of the 19 patients, 17 were tested for aPS/PT antibodies, with 12 (70.6%) testing positive. Of the 17 patients tested for aPS/PT antibodies, eight (47.7%) were positive for IgG aPS/PT, nine (52.9%) for IgM aPS/PT. The degree of thrombocytopenia was classified as follows: two patients (10.5%) had severe thrombocytopenia, eight patients (42.1%) moderate thrombocytopenia and nine patients (47.4%) mild thrombocytopenia. With regard to the treatment of thrombocytopenia, 11 out of the 19 patients (57.9%) required specific therapy. Two patients (10.5%) received a combination of corticosteroids and IVIG.

### 3.3 ITP and APS Cohorts Comparison

**FIGURE 2:**
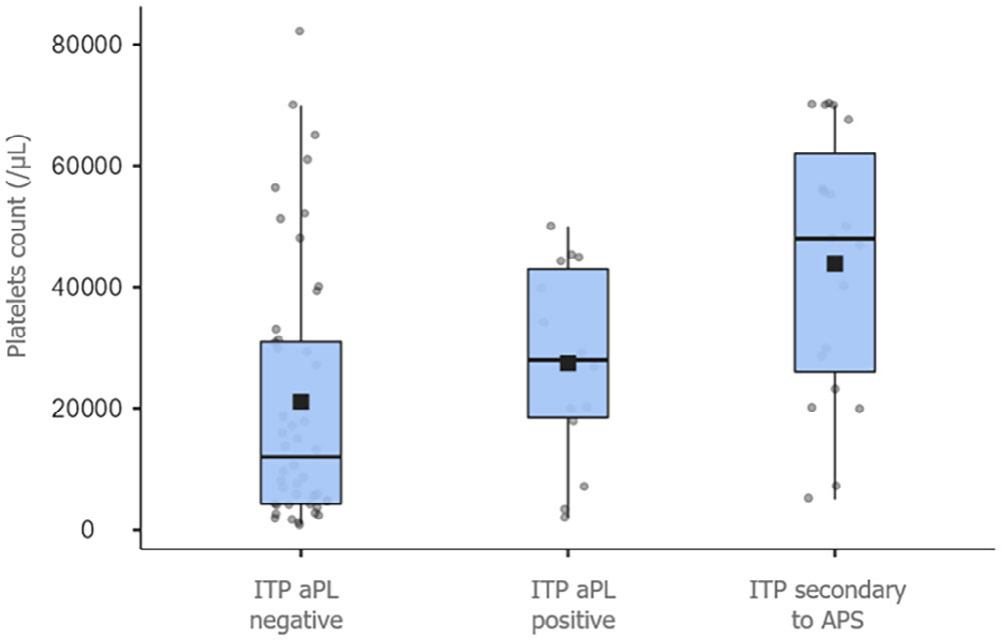
Mean platelet count in patients with aPL‐negative, aPL‐positive ITP, and ITP secondary to APS. Indication of platelets number in the indicated cohort of patients.

**FIGURE 3:**
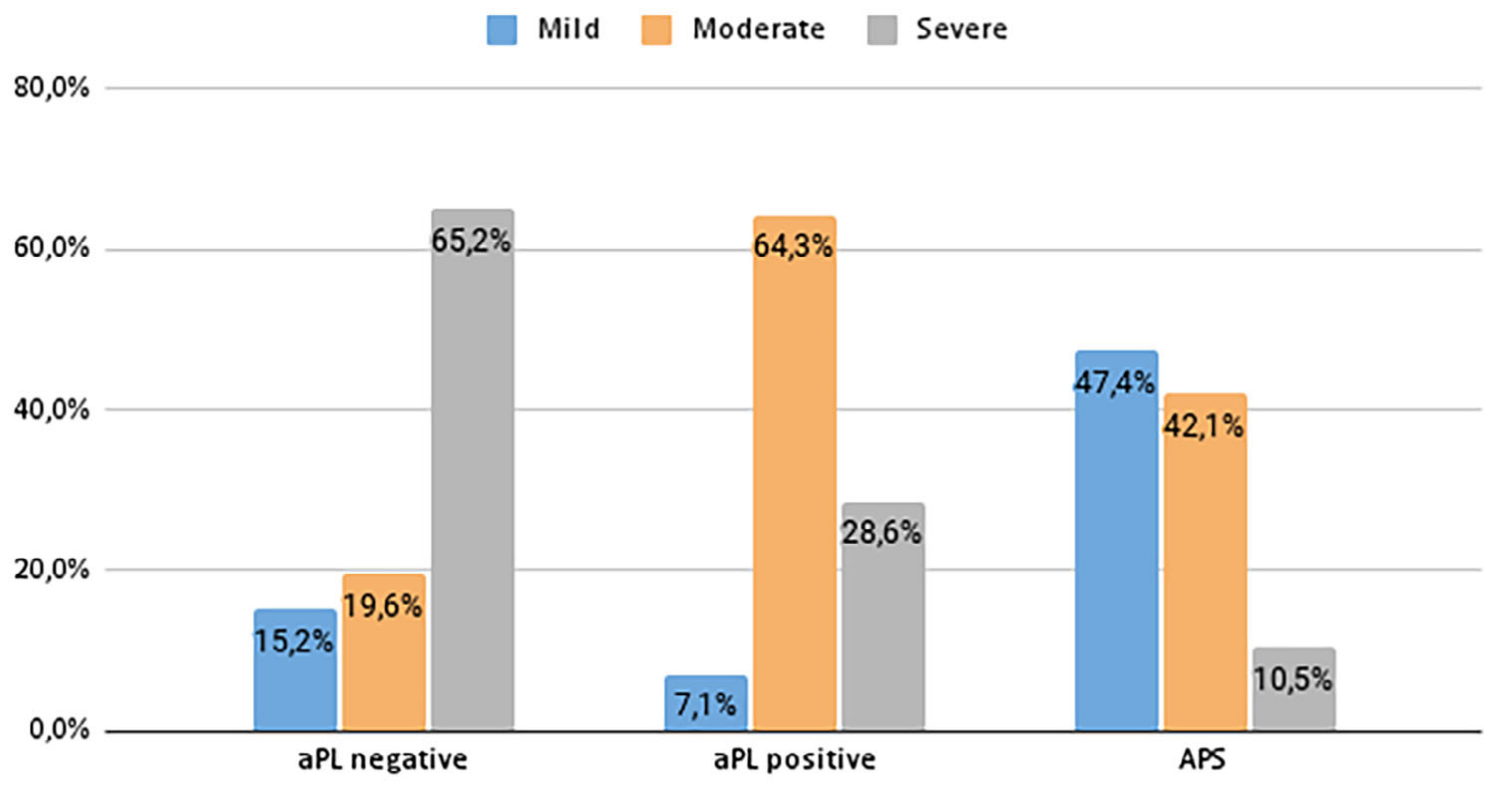
Severity of thrombocytopenia in patients with aPL‐negative, aPL‐positive ITP, and ITP secondary to APS. Distribution of platelet severity in the cohort of patients. The degree of severity of thrombocytopenia is classified as follows: mild (platelet count < 100,000 and ≥ 50,000/µL), moderate (< 50,000, and ≥ 20,000/µL) and severe (< 20,000/µL).

Based on aPL screening, patients with thrombocytopenia were divided into three distinct groups: ITP aPL‐negative, ITP aPL‐positive without clinical manifestations of APS, and ITP secondary to APS. A statistically significant difference in the severity of thrombocytopenia was observed between the groups. Patients with ITP secondary to APS had a higher mean platelet count (43,895 ± 21,947/µL) compared to aPL‐positive ITP patients (27,492 ± 16,327/µL, *p* = 0.07) and aPL‐negative ITP patients (21,130 ± 21,575/µL, *p* < 0.001), as illustrated in Figure 2. As shown in Figure 3, the majority of aPL‐negative ITP patients had severe thrombocytopenia (65.2%), while aPL‐positive ITP patients more frequently presented with moderate forms (64.3%), and APS patients predominantly exhibited mild thrombocytopenia (47.4%). This difference in the distribution of thrombocytopenia severity was statistically significant (*p* < 0.001). To further assess the impact of aPL positivity, we performed a multivariable linear regression analysis adjusting for age and sex to evaluate the independent effect of aPLs on platelet count.

In the subgroup analysis restricted to patients with primary ITP (excluding those with secondary ITP related to APS), aPL positivity was not independently associated with platelet count (*p* = 0.342).

Conversely, when analysing the entire cohort, including patients with secondary ITP due to APS, aPL positivity remained independently associated with platelet count (*p* = 0.006), even after adjusting for age (*p* = 0.841) and sex (*p* = 0.854). Neither age nor sex showed a significant association with platelet count in the adjusted model. Moreover, patients with ITP secondary to APS exhibited a reduced requirement for specific treatment for thrombocytopenia in comparison to aPL‐negative and aPL‐positive ITP patients (57.9% vs. 87.0% and 57.9% vs. 71.4%, *p* = 0.034). Furthermore, the necessity for second‐line therapy was markedly reduced in APS patients in comparison to aPL‐negative and aPL‐positive ITP patients (10.5% vs. 45.7% vs. 42.9%; *p* = 0.025).

## 4 Discussion

ITP is classified as primary or secondary, with the latter often linked to autoimmune disorders like APS. Thrombocytopenia is the most common haematologic manifestation in APS, occurring in 20%–50% of cases [21, 22]. Data from the Euro‐Phospholipid Project and the APS ACTION registry indicate a prevalence of thrombocytopenia of 29.6% and 19%, respectively, in patients with APS [23, 24]. Moreover, thrombocytopenia, initially classified as an extra‐criteria manifestation, has recently been included in the 2023 ACR/EULAR classification criteria for APS [3]. Although ITP and APS are regarded as distinct conditions, their interrelationship is intricate and the subject of ongoing debate. In 1985, Harris et al. first described the presence of aPL in patients with ITP [25]. The term'aPL‐associated thrombocytopenia' emerged to describe patients with positive aPL and thrombocytopenia, but without typical APS symptoms. Its clinical significance, especially in treatment, remains debated. Studies suggest aPL‐thrombocytopenia coexistence may elevate thrombotic risk and potentially lead to APS [26, 27, 28, 29, 30]. In particular, Diz‐Kücükkaya et al. reported that 45.1% of patients with ITP and persistent aPL positivity developed APS during the follow‐up period [27]. Hisada et al. demonstrated that the combination of aPL and thrombocytopenia is associated with a twofold increase in the risk of developing thrombotic events [28]. Despite emerging evidence, aPL‐positive ITP is still classified as primary ITP, potentially underestimating its clinical significance. This analysis aims to enhance understanding of ITP pathophysiology, especially where both bleeding and thrombotic risks coexist. Managing ITP increasingly requires balancing these risks. While TPO receptor agonists effectively raise platelet counts and reduce bleeding, they may increase thrombotic risk—particularly in patients with prothrombotic factors like aPL or APS features [31]. Clinicians must therefore adopt a personalised approach, considering bleeding history, thrombotic risk profile and underlying comorbidities.

This study found aPL positivity in 18.3% of ITP patients, mainly via LA (72.7%). LA detection can be challenging due to phospholipid‐dependent functional tests, but extra‐criteria antibodies like aPS/PT may assist. Literature suggests aPS/PT could serve as a surrogate for LA, especially in inconclusive or fluctuating cases [19, 32, 33]. In this study, 11.6% of patients tested positive for aPS/PT, some without aPL positivity required for APS classification. Findings suggest incorporating aPS/PT in ITP diagnosis could refine subgroup identification. aPL positivity, including aPS/PT, was significantly linked to thrombocytopenia severity (*p* = 0.006); 64.3% of aPL‐positive patients had moderate thrombocytopenia, while 65.2% of aPL‐negative patients had severe thrombocytopenia. Although first‐ and second‐line therapy needs did not differ significantly, aPL‐positive ITP patients required fewer steroids and IVIG than aPL‐negative patients (21.4% vs. 65.2%, *p* = 0.003). In the APS cohort, thrombocytopenia affected 15.9% and was mostly mild/moderate. LA was the most prevalent antibody (84.2%), and aPS/PT was positive in 70.6%. Severe thrombocytopenia was less common in ITP secondary to APS (10.5%) versus aPL‐positive ITP (28.6%) and aPL‐negative ITP (65.2%, *p *< 0.001). APS‐related ITP patients required thrombocytopenia treatment less often (57.9% vs. 71.4% aPL‐positive, 87.0% aPL‐negative, *p* = 0.034) and needed fewer second‐line therapies (10.5% vs. 45.7% aPL‐negative, 42.9% aPL‐positive, *p* = 0.025). Screening for aPL represents a useful tool to identify a distinct ITP phenotype. Although these cases are classified as primary ITP in the absence of other clinical manifestations of APS, they exhibit clinical features that are intermediate between aPL‐negative ITP and ITP secondary to APS. It is possible that aPL‐positive ITP may evolve into APS, particularly if thrombotic complications arise. In our patient cohort, we did not observe any thrombotic or vascular events. This absence may, at least in part, be attributable to the careful monitoring of thrombotic and obstetric risk factors, along with the implementation of primary prevention strategies when deemed appropriate. This emphasises the necessity for careful management and monitoring. Moreover, the introduction of the aPS/PT antibody test as an additional criterion could improve the diagnostic accuracy for both ITP and APS, especially in patients who test negative for the antiphospholipid antibodies included in the diagnostic criteria. Our study underscores the remarkable clinical heterogeneity of ITP, revealing that, in a subset of patients, it may closely mirror features typically associated with APS. These findings raise the possibility that ITP and APS, rather than being entirely distinct entities, may at times represent divergent clinical expressions of a shared underlying systemic autoimmune process. Recognizing this intersection is not merely of academic interest—it carries significant clinical weight. By identifying patients who exhibit overlapping features of ITP and APS, particularly those prone to thrombotic complications or resistant to standard therapies, clinicians are better equipped to anticipate complex disease trajectories. This recognition paves the way for a truly individualised approach to care—one that judiciously balances the need for thromboprophylaxis with the imperative to mitigate bleeding risks, and that adapts immunosuppressive strategies accordingly. Such precision in diagnosis and management is essential in the era of therapies that may exacerbate thrombotic tendencies, such as TPO receptor agonists [34]. Ultimately, this nuanced understanding enhances clinical decision‐making, reduces the risk of adverse outcomes and affirms the importance of tailoring treatment to the multifaceted nature of each patient's disease. Building on these insights, testing for antiphospholipid antibodies (aPL) may serve as a valuable tool for patient stratification, enabling a more personalised therapeutic approach. For instance, TPO receptor agonists may be more suitable for aPL‐negative patients, while spleen tyrosine kinase (Syk) inhibitor Fostamatinib [35] could represent a safer and more effective option for aPL‐positive individuals, given their potential thrombotic risk profile. While the study provides valuable insights into the ITP–APS overlap, certain limitations should be noted. Restricting inclusion to APS patients with platelet counts <100,000/µL may limit generalisability. Stratification by aPL profiles is informative but subject to assay variability and evolving definitions, especially for non‐criteria antibodies like aPS/PT. Despite efforts to reduce confounding (e.g., excluding patients who received IVIg before aPL testing), residual biases related to treatment history and disease duration may persist. Prospective, multicentre studies with standardised protocols are warranted to validate these findings.

## Author Contribution

S.C., G.R., M.P, I.R. and C.B. analysed ITP cohort; M.R, A.B., I.C, D.G. analysed APS cohort; B.M. assessed APS antibodies; S.S. and A.M. conceptualised the work and reviewed data. F.P and M.DG reviewed the manuscript. S.C. and A.M wrote the manuscript.

## Conflicts of Interest

The authors declare no conflicts of interest.

## Data Availability

The data that support the findings of this study are available on request from the corresponding author. The data are not publicly available due to privacy or ethical restrictions.
